# Non-destructive methods for fruit quality evaluation

**DOI:** 10.1038/s41598-021-87530-2

**Published:** 2021-04-08

**Authors:** Ana-Maria Bratu, Cristina Popa, Mihaela Bojan, Petre Catalin Logofatu, Mioara Petrus

**Affiliations:** National Institute for Laser, Plasma and Radiation Physics, 409 Atomistilor St., PO Box MG-36, 077125 Bucharest, Romania

**Keywords:** Plant biotechnology, Plant development, Plant evolution, Plant hormones, Plant physiology

## Abstract

This work studies the evolution in time of several varieties of apples with application in quality storage maintenance. Two different methods were used to evaluate long-stored apples for better sorting and degradation assessment. The first method was laser photoacoustic spectroscopy for the detection of ethylene and ethanol compounds from the internal atmosphere of apples. The second method was multispectral imaging that measures the image and the spectrum combined and also can be used to address features such as ripening and external defects. The experiments showed that, the ethylene value decreases and the value of ethanol increases, which sometimes we may associate with a drift of the images toward darker tones, because the apple is slowly degrading. Non-invasive, real-time inspection can reveal when the degradation process begins, improving the capability of sorting, maintaining their quality and storability.

## Introduction

Apples are one of the most cultivated and consumed fruits in the world. It is an important and loved fruit. Since apples are climacteric fruits, the ripening of these fruits must be delayed for long-term storage^1^. Low temperatures and controlled atmosphere are generally used for stored fruit in order to delay ripening and to extend the storage period^2^. In maintaining high-quality of apples, sorting is important because it classifies the fruit according to parameters such as size, diameter, length, and shape, thus defining sorting classes for many fruit varieties. Under proper storage conditions, apples can be stored for up to 12 months^3^.

Fundamental requirements for extending the time that fruits can be stored and marketed is to ensure an adequate external environment and to investigate the internal environment of the commodity. Light and temperature are the main external environmental factors that can affect fruits storage^4^. The internal environment is defined by a complex of gases and volatiles that can diffuse through fruit tissue controlling the development of the commodity^5^. These volatiles are mostly esters, alcohols, aldehydes, ketones and ethers^6^. In apples, ethylene is present in high concentration during the development period^7–9^, but decreases as the fruit matures, following a stage in which the other volatiles such as alcohols and esters increases^10,11^. All the volatile compounds are of great importance and can be used to determine the optimum maturity stage^7^. Their concentration differ among varieties^12,13^ and can be influenced by different factors and conditions during and after the harvest^14^. The production of volatile compounds is a critical factor and it is important to evaluate their interaction with ethylene in postharvest situations.

Exploration of new relationships between ethylene and ethanol can be of great help in the processof plant development andin improving crop yield by delaying aging and maintaining fruit quality. The role played by ethanol in this relationship remains largely unexplored.

In fruit, ethylene is emitted naturally in the process of ripening or may be produced when plants are injured in some way^15^. This phytohormone is synthesized in great quantities during the late-ripening stage and is involved in a wide range of processes, including fruit ripening, abscission, senescence and responses to biotic and abiotic stresses^16,17^. All these processes are characterized by an increase in ethylene concentration in the fruit developmental cycle. Ethylene is biologically active at very low concentrations from 0.01 to 1.0 parts per million (ppm)^18^. Depending on the type and reactions, lower or higher sensitivities were observed. Tomatoes and apples are some of the climacteric fruit that can generate tens of ppm of ethylene. The possibility to control the effect of ethylene brought new ways to improve the postharvest fruit quality^19^.

Ethanol is also naturally produced by fermentation process of fruit sugars by yeast. In general, ethanol production (through anaerobic metabolism) appears when concentrations of O_2_ are very low. This can occur when levels of external O_2_ are reduced because of the increased resistance to the diffusion of O_2_ into the fruit. Development and maturation of fruits cause changes in the levels of O_2_ and CO_2_ inside them^20^. These changes are usually represented by a net reduction in O_2_ to CO_2_ ratio within the fruit and results in the accumulation of ethanol. Ethanol detection in an internal atmosphere of fruit can provide an additional measure of maturity.

Both external defects and internal physiological changes of apples are important factors in the post-harvest, sorting and storage period. Rapid identification of defective fruits is beneficial for reducing unnecessary storage and avoiding quality degradation. Over the years, considerable efforts have been made to develop non-destructive sensing techniques for quality evaluation of apples, mainly spectroscopic and imaging. For quality evolution of internal atmosphere of apples the most investigated gaseous phytohormone was ethylene known to regulate growth and development process^21^. The quantitative and qualitative analysis of ethylene and others volatile compounds has been analyzed using different sensitive methods. Among these methods are to be mentioned gas chromatography (GC) detection, electrochemical sensing and optical gas sensing^22^. Major volatile compounds were typically detected using GC or GC–MS because are easy to operate, offer fast measurement, and components can be separated from complex mixtures^23^. However GC has limited sensitivity, requires a pre-concentration and a performing system can be found at a very high cost^24^. Electrochemical sensors were used for ethylene detection^25^ but lately they are used for a wide range of compounds^26^. This kind of sensors are inexpensive, accurate and present repeatable results but they are very sensitive to interfering gases, temperature and humidity changes, and require limited temperature range and oxygen to operate correctly. Optical sensors are successfully utilized for detection of gas components. One of the most popular optical gas sensors is infrared laser-based gas sensors that converts electromagnetic radiation energy into electrical signals. CO or CO_2_ lasers are preferentially used to selectively excite the specific molecules and in association with detection techniques^27–30^, such as photoacoustic spectroscopy results infast detection at low concentrations. Laser photoacoustic spectroscopy (LPAS) techniques offers detection limits that are very hard to achieve with other methods. In our laboratory was developed a CO_2_ laser-based photoacoustic spectroscopy set-up with high sensitivity and good selectivity, very fast response time (seconds) and real-time monitoring. This instrument is widely used to measure trace gases at ppm or ppb level and the reported ethylene measurements from fruit made using this technique are perhaps the most reliable measurements available^31^.

Information about external defects is important for sorting the mature and over mature fruits with the best attributes. External defects were traditionally detected on manual inspection but it is a tedious and laborious process and disposed to human errors. In recent years machine vision was used for automatically inspection of apples, but it less efficient in detecting surface defects. Hyperspectral and multispectral imagingare similar technologies that have advantages over conventional machine vision imaging methods. They both have been largely used for defect detection of apples. However, multispectral imaging cost-effectiveness clearly has an advantage over hyperspectral imaging.

In what follows, we are trying to bring the CO_2_ laser photoacoustic spectroscopy together with multispectral imaging as a first attempt to investigate both the internal and external differences that appear in apple life over a period of 35 days.

The first objective of this work was to observe the effects of ethylene and ethanol emission from the apples (Golden, Gala, Granny Smith and Sarkrimson apples) under aerobic conditions at room temperature.The production of ethylene and ethanol in apples was investigated as a result of diverse physiological processes that varies due to crop maturity.

The study will also focus on identifying external defects as quickly as possible as one of the most important factors for fruit inspection. The external appearance of fruit was investigated using multispectral imaging, a technique that provides information hidden to the human eye and can also be automated.

Using these two methods, we can make a connection between the reflectivity of the apples and the internal ethylene and ethanol gas emission of apples that provide helpful guidelines for minimizing deterioration, keeping the overall quality, and lengthening the shelf life.

This study presents two techniques that support innovation in postharvest management of fruit that helps to reduce postharvest losses during marketing by preventing deterioration and improve the final overall quality of commodities.

## Results

In the present paper we have performed analyses on Malus domestica Golden Delicious, Gala, Starkrimson and Granny Smith. Commercially grown apple samples were procured from a local supermarket (Magurele, Ilfov, Romania) with the specifications: Country of origin: Romania.

Twenty control apples (5 apples of each type) were studied on every 7 days at room temperature for a period of 35 days. The objective was to determine the quality attributes of apples according to the number of days using simple, but precise methods, capable of investigating the external and internal characteristics.

**Figure 1:**
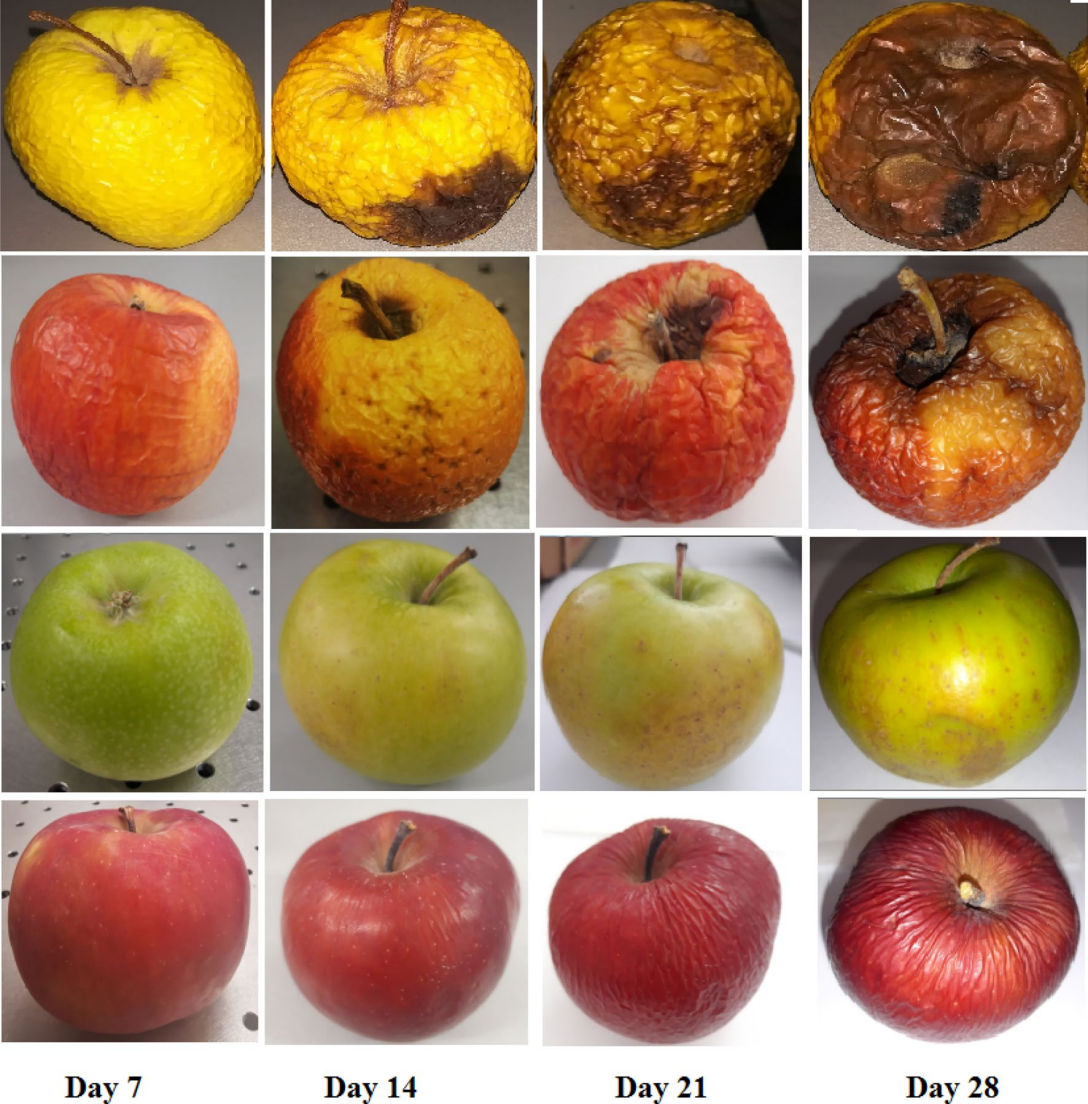
Pictures representing the evolution of a Golden, Gala, Granny Smith and Starkrimson apples over 35 days. External appearance of apples that continues to degrade presenting speckle and wrinkles.

At the beginning they were normal apples (without defects) and at the end of the study they were already rotten, presenting speckle and wrinkles (see Fig. 1).

### Protocol for quantification of ethylene and ethanol

Before starting the experimental study we calibrated the PA system using ethylene as reference gas because its absorption coefficients are accurately known at CO_2_-laser wavelengths and presents negligible adsorption on the cell walls. For our experiments, calibration of the system is made using certified mixture containing 0.96 ppmV ethylene in pure nitrogen that is introduced in the PA cell at a total pressure of approximately 1027 mbar and a temperature of 23 °C. Ethylene exhibits characteristic absorption peak at the 10P (14) laser transition at 949.49 cm^−1^, where it has an absorption coefficient of 30.4 cm^−1^ atm^−1^. According to the calibration of the system, we measured the responsivity R = 332 cmW/V.

After the calibration, the gas from the cell is evacuated and the cell is cleaned with a flux of pure nitrogen at atmospheric pressure. When the system becomes clean, the cell is filled with pure nitrogen at atmospheric pressure and the limiting PA background signal is measured at resonance frequency. The PA background signal of 2.7 µ V/W was observed that is equivalent to an absorption coefficient of 2.7 × 10^–8^ cm^−1^, or an ethylene concentration of about 0.9 ppbV.

Once the system has been calibrated we can start measuring the samples.

All apple fruit used in these measurements were stored at room temperature for subsequent use. The fruit were introduced into the glass cuvette with the volume of 150 cm^3^ and then connected to the PA cell. Prior to the start of the detection process, we evacuated thoroughly the previous gas mixture from the entire handling system, including the PA cell, and then we cleaned the system for a few minutes with nitrogen. After a second vacuum cleaning, the measurements were started.

The fruit samples were flushed with synthetic air flow (20% oxygen and 80% nitrogen with impurities: hydrocarbons max. 0.1 ppmV and nitrogen oxides max. 0.1 ppmV) at atmospheric pressure (1027 mbar) and the resulting gas from the glass samples was transferred in the cell and analyzed. Concentrations of ethylene and ethanol that are normally produced by apple in normal aerobic conditions (synthetic air flow) were observed over time (day 1, 7, 14, 21, 28 and 35).

The assessment of ethylene and ethanol were performed for specific CO_2_ laser lines. For ethylene detection 10P (14) laser line was selected where we have the maximum absorption coefficient of 30.4 cm^−1^ atm^−1^^29^. For ethanol was selected 9R (22) laser line where we have the maximum absorption coefficient of α = 4.081 cm^−1^ atm^−1^^32^.

### Ethylene emission

Long-term experiments provide insight into the processes that produce changes in mature fruits and helped us to understand the significance of ethylene that differ in the rate of production under non-stressful conditions. The amount of ethylene emitted by Golden, Gala, Granny Smith and Starkrimson apples was observed over 35 days. The apples were chosen about the same size and weighing approximately 140 g. Ethylene emission from each apple was established by introducing apple into the cuvette and flushed with synthetic air flow at atmospheric pressure and the resulting gas from the glass samples was transferred in the cell and analyzed for 300 s.

A decrease in ethylene can be observed for all four varieties of apples. The Golden and Gala varieties showed the same behavior of the variation of the ethylene concentration and the highest values of ethylene at the beginning of the measurements. Thus, the two varieties presented a slight decrease in the concentration of ethylene until day 14, followed by a very large decrease from day 14 to day 21, and a slight decrease in the concentration of ethylene from day 21 to day 35. We can observe that from the day 28 the ethylene is minimal which means that the respiration rate of apples is very low.

**Figure 2:**
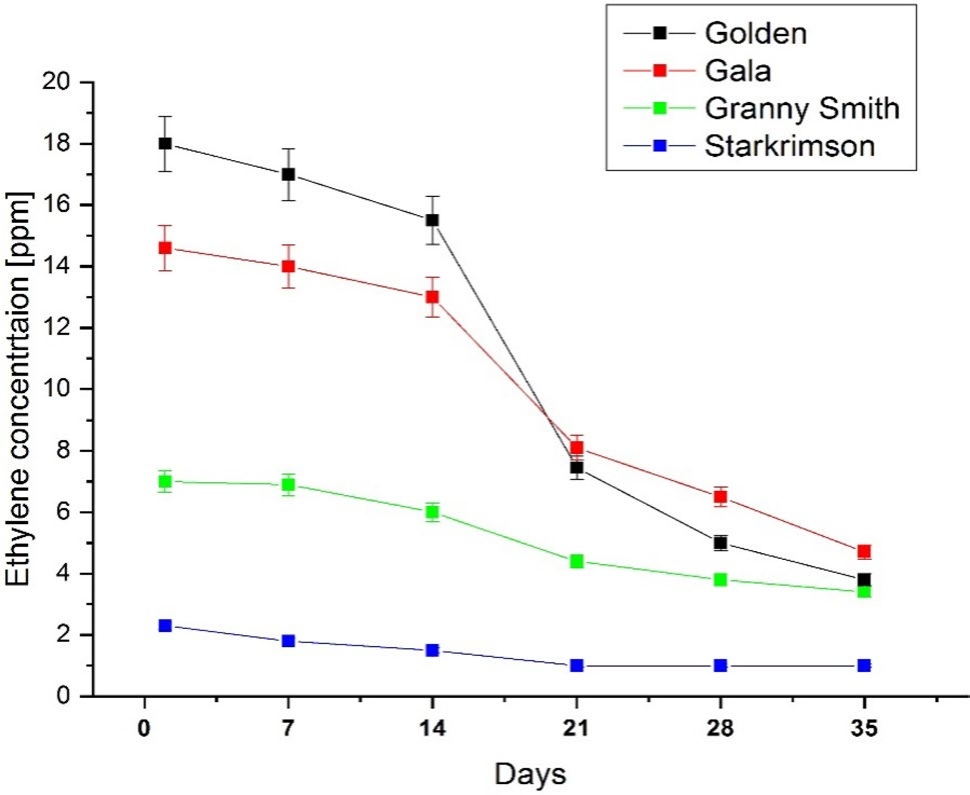
The variation of the ethylene concentration over a storage period of 35 days at room and atmospheric pressure. A decrease in ethylene can be observed for all four varieties of apples, especially from day 21. The Golden and Gala varieties manifested the same behavior of the variation of the ethylene concentration and the highest values of ethylene at the beginning of the measurements.

The Granny Smith and Starkrimson varieties both exhibit the same behavior in varying the concentration of ethylene. Compared to the Golden and Gala varieties, recorded lower values of ethylene concentration from the beginning of the measurements. However, as can be seen from Table 1 and Fig. 2, these apple varieties also recorded a greater decrease in ethylene concentration on day 21 compared to the first 14 days, followed by a slight decrease. This two varieties of apples had the lowest respiration rate starting from the day 21.

**Table 1:** Ethylene production of apples over 35 days.

Days	Mean ethylene concentration of Golden Apples (ppmV)	Mean ethylene concentration of Gala Apples (ppmV)	Mean ethylene concentration of Granny Smith Apples (ppmV)	Mean ethylene concentration of Starkrimson Apples (ppmV)
Day 1	18	14.6	7	2.3
Day 7	17	14	6.9	1.8
Day 14	15.5	13	6	1.5
Day 21	7.45	8.1	4.4	1
Day 28	5	6.5	3.8	1
Day 35	3.8	4.7	3.4	1

At room temperature conditions, Golden and Gala apples became wrinkled and small brown patches appeared after only 7 days. Granny Smith and Starkrimson withstood more at room temperature starting to present wrinkles and brown patches after 14 days. The process of fermentation started earlier in the Golden and Gala varieties being accelerated by the high production of ethylene.

Changes occurring in apples over a 35-day period follow a sequence of events, starting with the softening of the flesh and skin, the appearance of wrinkles and soft brown spots patches, and the weight loss of the apples.

### Ethanol emission

The ethanol emission was measured at the same time with ethylene by changing the laser line. A similar approach was observed in the ethanol emission from all apples with low initial concentration and a significant increase at the end of the 35 days.

**Figure 3:**
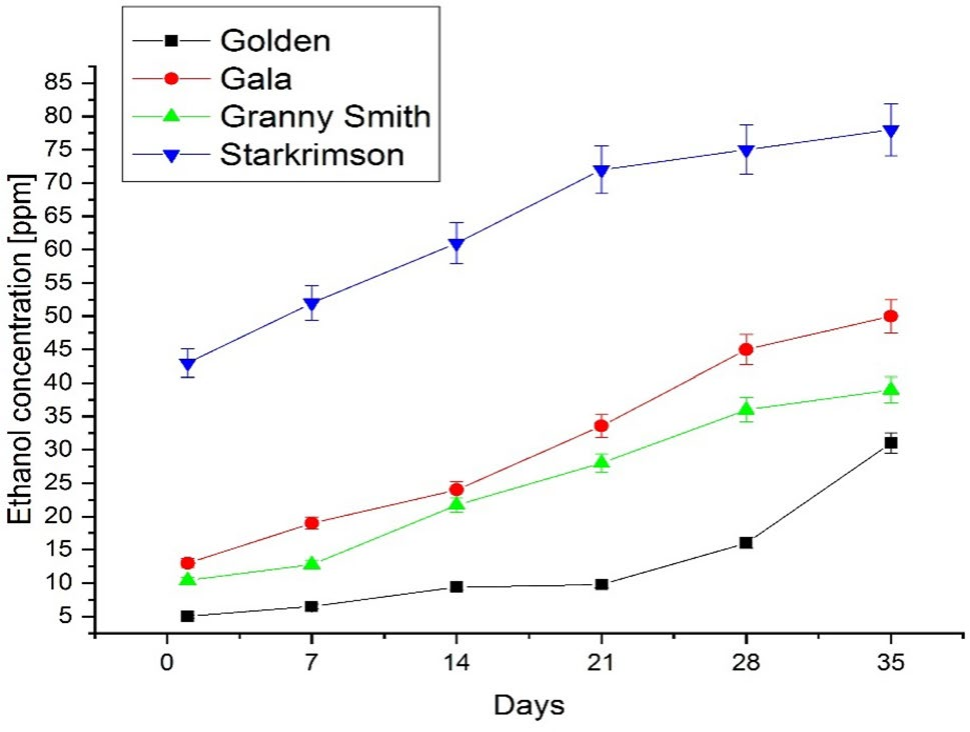
Ethanol concentrations obtained using the CO_2_ LPAS system from the respiration of four varieties of Golden, Gala, Granny Smith and Starkrimson apples over a period of 35 days. The variation of the ethanol concentration from apples over a storage period of 35 days manifested an increase in ethanol emission for all four varieties of apples especially for Gala and Starkrimson.

In the first 14 days, ethanol emissions showed a slow increase, for Golden, Gala, and Granny Smith fruit a greater increase being visible from day 21. For Starkrimson apple we can say that the increase of ethanol was almost constant every week, but present in highest concentration compared to other varieties investigated. A major accumulation of ethanol in all apples was observed on day 35 when fruits were rotten with brown spots patches and wrinkles (see Table 2 and Fig. 3).

**Table 2:** Ethanol production of apples over 35 days.

Days	Mean ethanol concentration of Golden Apples (ppmV)	Mean ethanol concentration of Gala Apples (ppmV)	Mean ethanol concentration of Granny Smith Apples (ppmV)	Mean ethanol concentration of Starkrimson Apples (ppmV)
Day 1	5	13	10.4	43
Day 7	6.5	19	12.8	52
Day 14	9.4	24	21.7	61
Day 21	9.8	33.6	28	72
Day 28	16	45	36	75
Day 35	31	50	39	78

From Fig. 3 it can be seen that all four apple varieties showed a significant increase in ethanol concentration starting on day 14. The highest ethanol concentrations were determined in the Starkrimson variety and the lowest concentrations in the Golden variety. These varieties (see Fig. 2) presented an inverse behaviour in the case of ethylene concentration, the Starkrimson variety had the lowest values of ethylene, and the Golden variety had the highest values of ethylene concentration. It can be remarked that from the day 28 Golden apples still presented an increase in ethanol emission compared to Gala, Granny Smith and Starkrimson that no longer had an obvious increase rate.

From Figs. 2 and 3 it can be seen that the four varieties of apples exhibit the same behaviour, in the first 14 days the ethylene concentrations were high and the ethanol concentrations were low, and from day 14 the ethylene concentration decreases and the ethanol concentration begins to increase.

The Starkrimson apple variety, as can be seen in Figs. 2 and 3, had the lowest values of ethylene, but the highest values of ethanol concentration, which means that after harvesting the ripening process stagnates and begins the process of fermentation which is very low in the absence of ethylene.

Stored at room temperature, the four varieties of apples continue to ripen in the first 14 days, after which the fermentation process begins.

**Figure 4:**
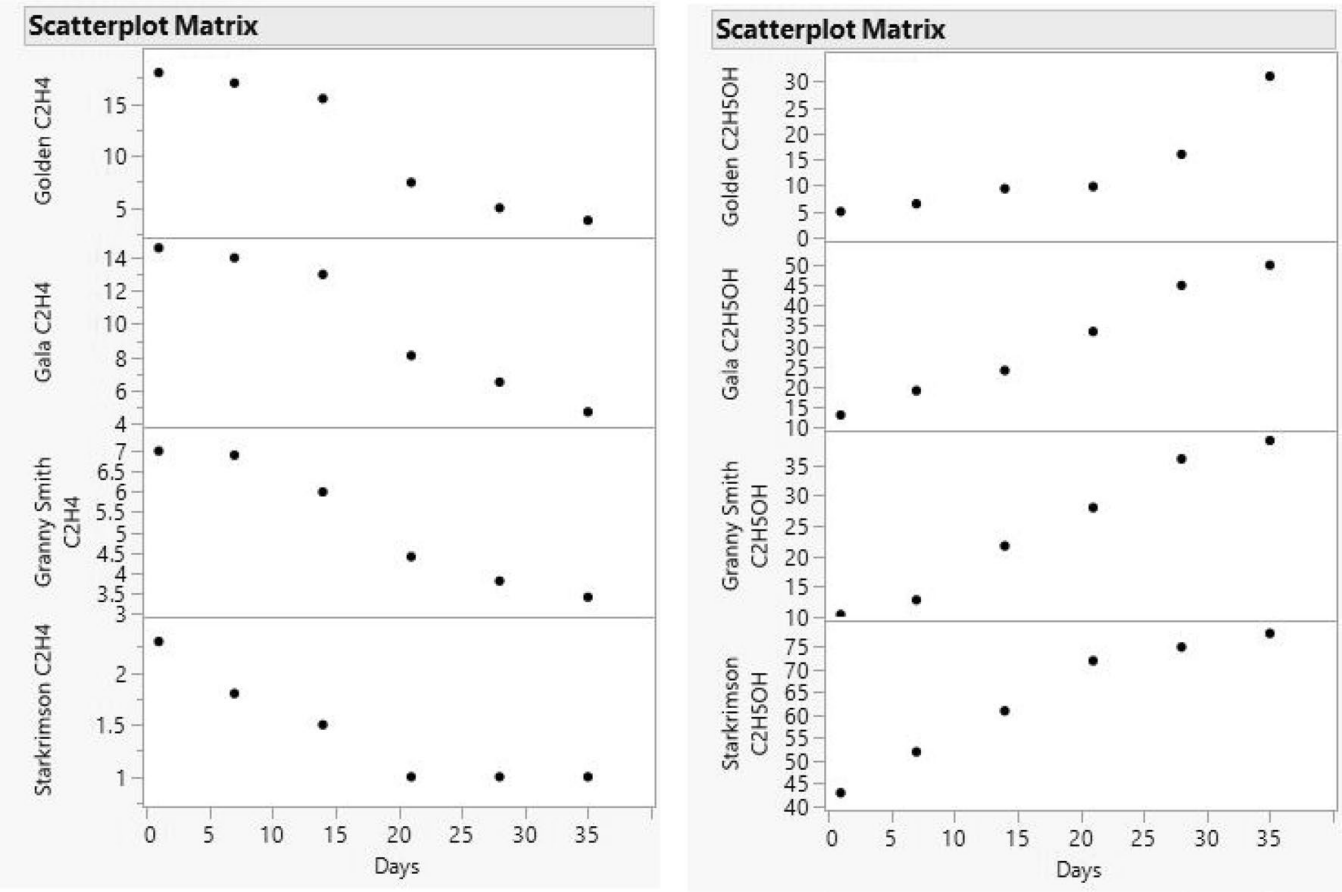
Scatter plot of ethylene (left) and ethanol (right) concentrations for apple varieties Golden, Gala, Granny Smith and Starkrimson over a period of 35 days, at an interval of 7 days.

The behaviour of ethylene and ethanol concentrations in the four apple varieties can be seen in Fig. 4, where the ethylene concentrations decrease in the four apple varieties, the decrease being pronounced after the first 14 days, reaching a plateau between days 21 and 35, and the ethanol concentration begins to increase significantly from day 14.

### Evolution in time of the histograms for color channels and spectral filters using multispectral imaging

The external defects on apples were investigated using a multispectral imaging system based on 8 spectral filters, namely the evolution in time of the histograms of the images corresponding to the various filters was monitored for several days.

**Figure 5:**
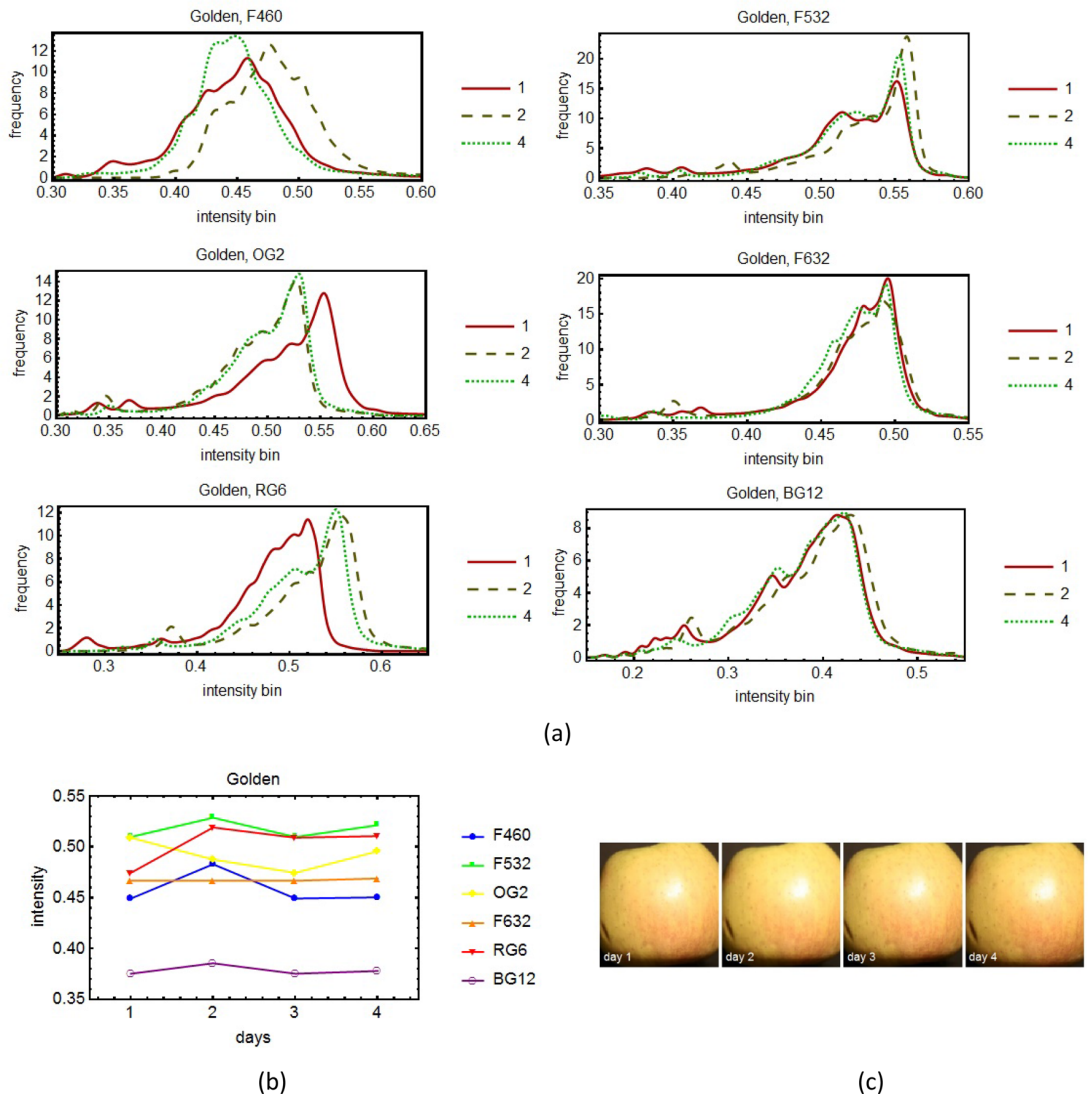
The time sequence of spectral evolution for the apple variety Gala: the histograms of the pictures taken for three days (the first, the middle and the last) for 6 spectral filters (**a**). In (**b**) it is shown a more quantitative and global representation of the evolution in time for all the days of the experiment. For conformity, in (**c**) are shown the pictures of the apple taken in white light, without the spectral filters, for all the days we monitored the apple.

**Figure 6:**
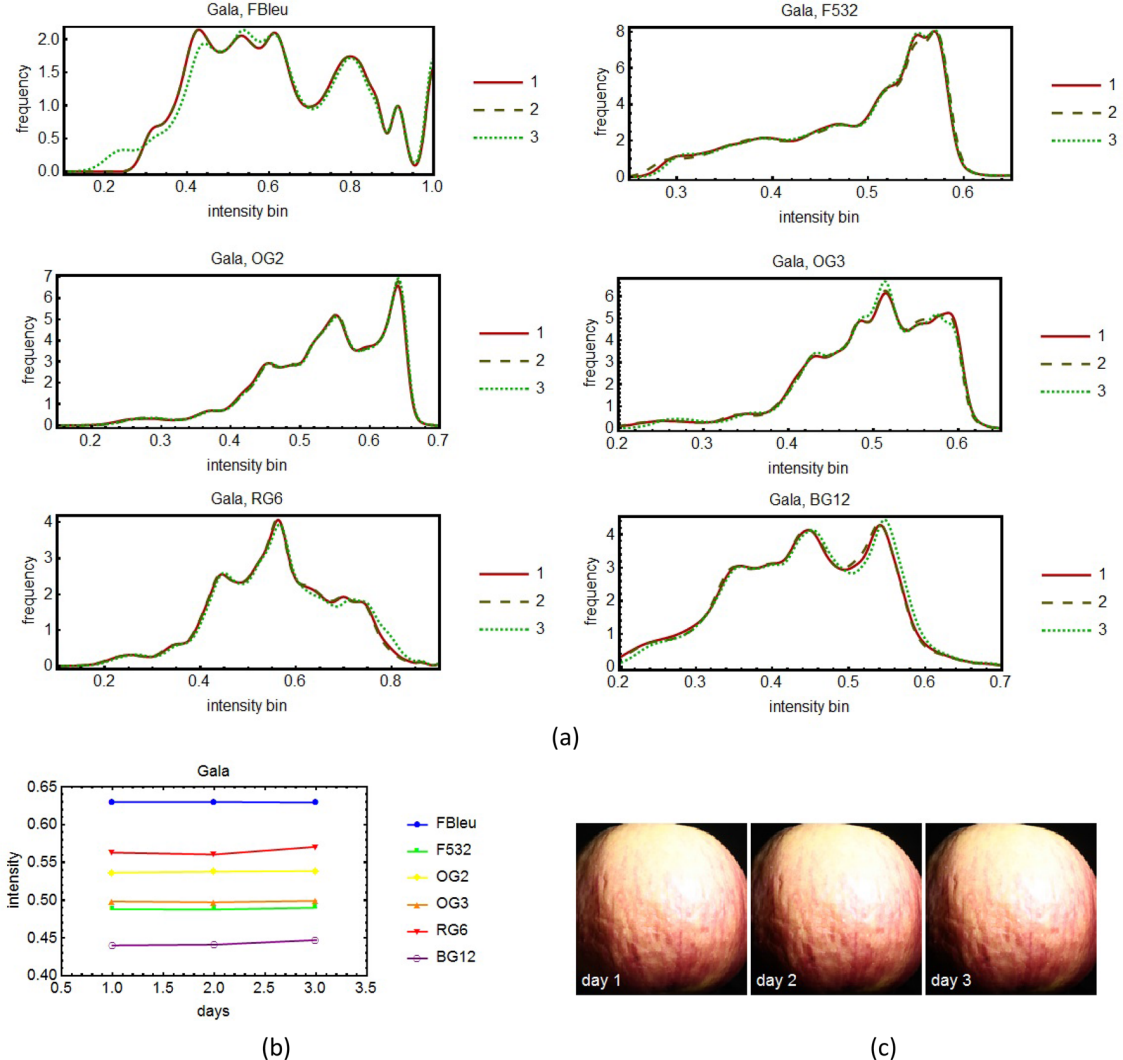
The time sequence of spectral evolution for the apple variety Gala: the histograms of the pictures taken for three days (the first, the middle and the last) for 6 spectral filters (**a**). In (**b**) it is shown a more quantitative and global representation of the evolution in time for all the days of the experiment. For conformity, in (**c**) are shown the pictures taken in white light, without filters of the apple for the same days as in (**a**).

**Figure 7:**
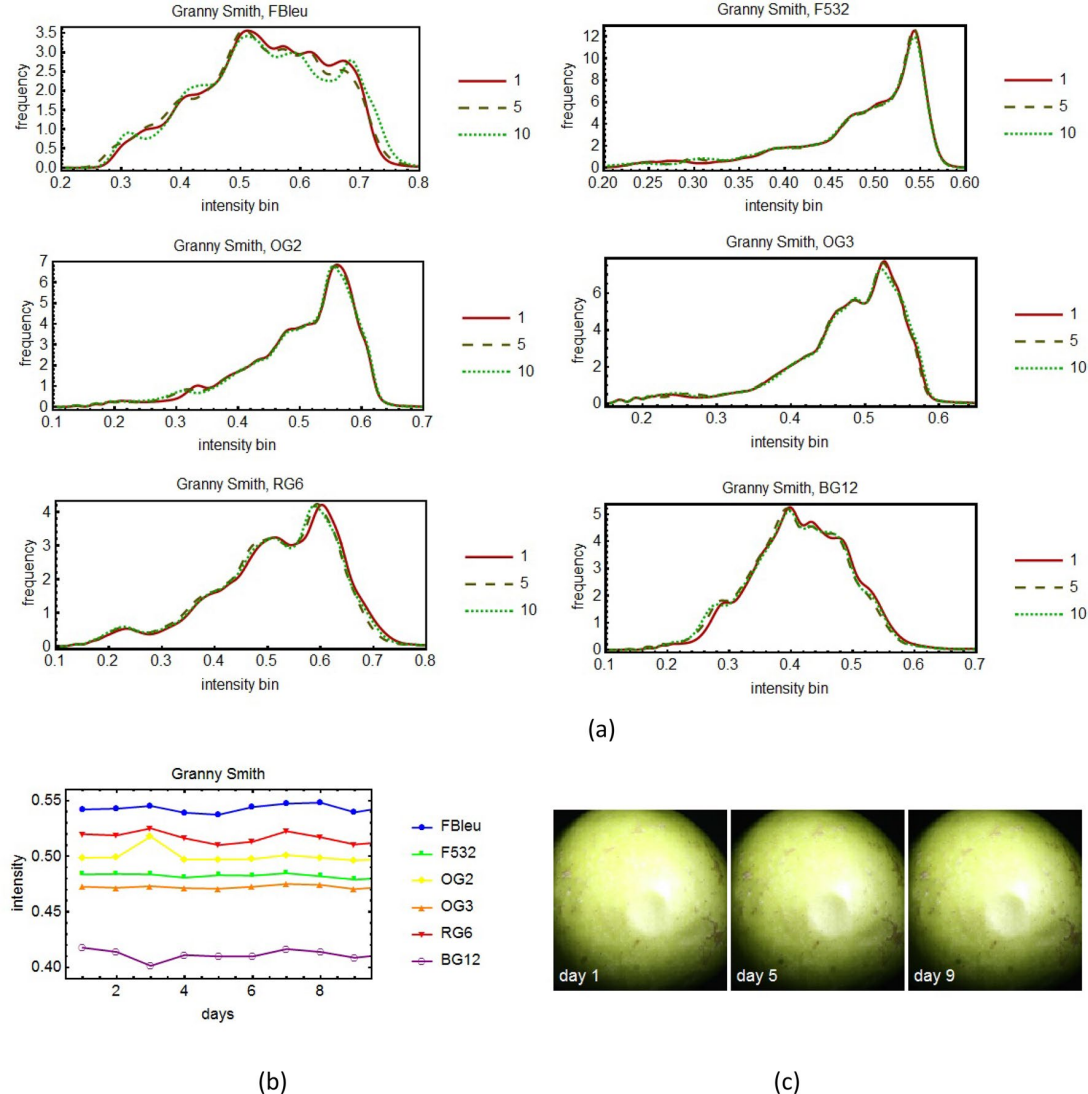
The time sequence of spectral evolution for the apple variety Granny Smith: the histograms of the pictures taken for three days (the first, the middle and the last) for 6 spectral filters (**a**). In (**b**) it is shown a more quantitative and global representation of the evolution in time for all the days of the experiment. For conformity, in (**c**) are shown the pictures taken in white light, without filters of the apple for the same days as in (**a**).

**Figure 8:**
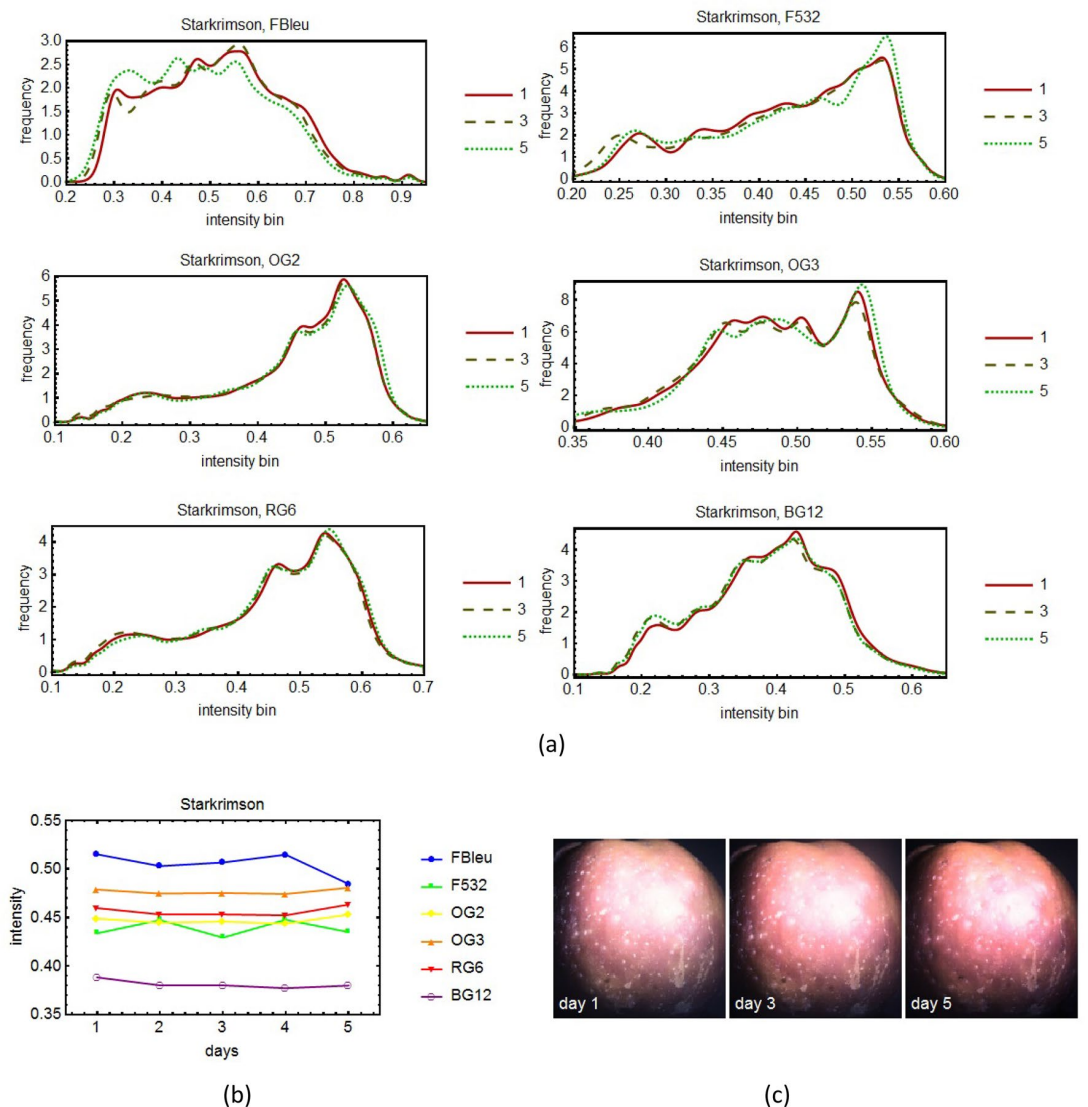
The time sequence of spectral evolution for the apple variety Starkrimson: the histograms of the pictures taken for three days (the first, the middle and the last) for 6 spectral filters (**a**). In (**b**) it is shown a more quantitative and global representation of the evolution in time for all the days of the experiment. For conformity, in (**c**) are shown the pictures taken in white light, without filters of the apple for the same days as in (**a**).

**Figure 9:**
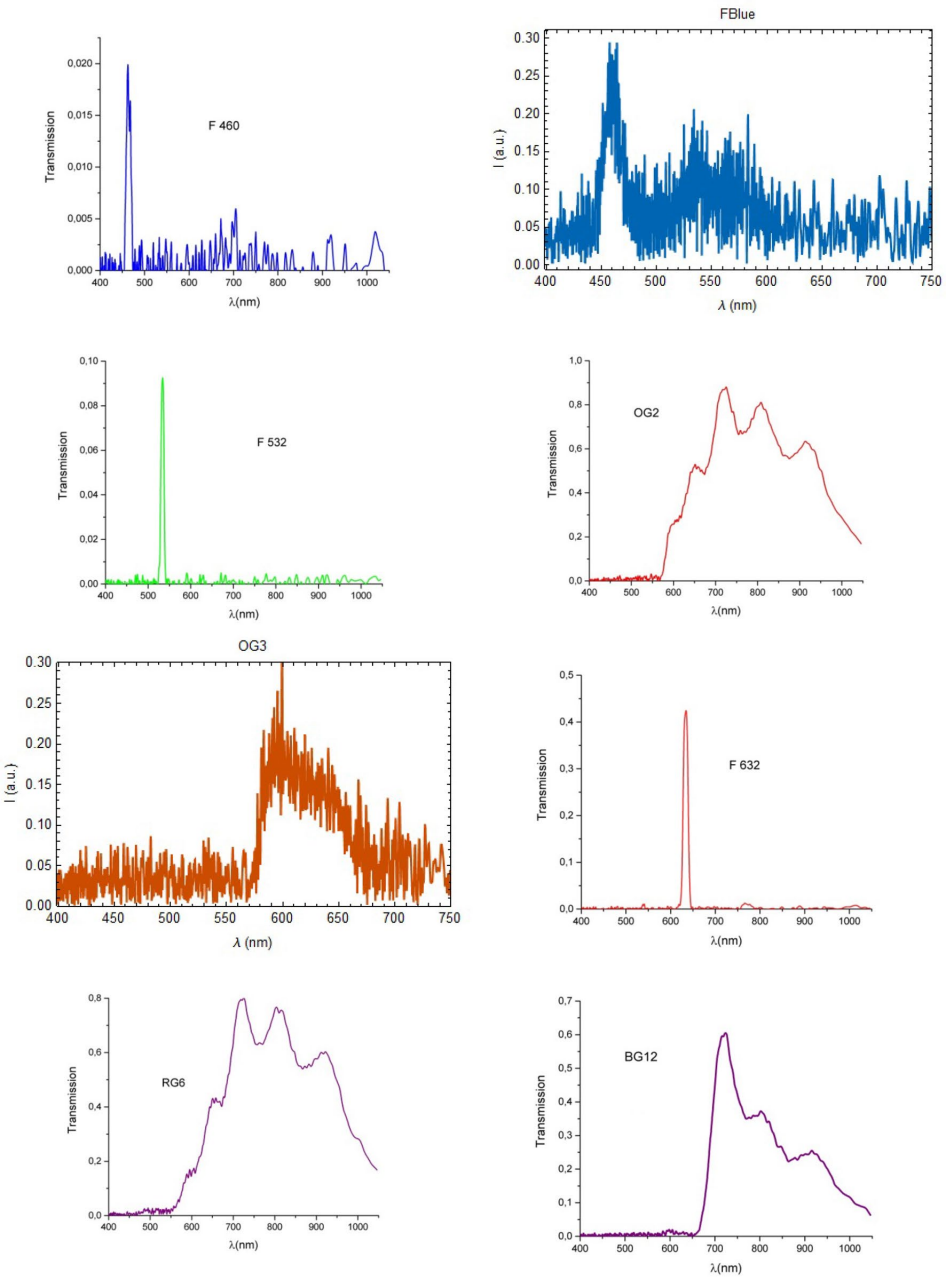
The characteristics of the spectral filters used in the multispectral imaging experiments.

In Figs. 5, 6, 7 and 8 the results of our multispectral imaging experiments are illustrated. For analysis we chose a large central region of the sample image, (Figs. 5, 6, 7, 8c). In order to be able to follow the evolution in time of the same point of the apple surface, some elementary techniques of Digital Image Correlation (DIC) using the Fast Fourier Transform (FFT) were applied^33^. Each point of the apple was monitored separately. In order to extend the dynamic range of the photos a little, we used the fact that the photographic sensor has a sensitivity that can be approximated by the sigmoid logistic function, which is linear in the middle. In order to interpret the multispectral imaging data, some data about the spectral filters used in the imaging experiments is necessary. For the filters RG6 and BG12 some data may be found in^34^. In Fig. 9 the spectra of the filters used is shown.

In the spectral histograms (Fig. 5, 6, 7, 8a) only three days from the period of monitoring are shown, the first, the middle and the last. If more days were shown, it would have been very difficult to see clearly the data. However, in the quantitative global plots (Fig. 5, 6, 7, 8b), in which an average of the intensity of the image of the apples are shown, all the days are present.

Unfortunately, various practical impediments prevented us from having uniform experimental conditions for all the studied cases. The differences between the histograms of consecutive days were expected to be slight, and for the most part they were. One clear exception is apple variety Golden. We tried to discern patterns of evolution in time but for the most part we were unsuccessful. We had one small success with the apple variety Granny Smith (Fig. 7). For this apple we benefited from more extensive experimental data. It turns out there is a slight overall tendency toward darker tones for this particular apple.

In some cases one day yielded out of the ordinary data. For example, see day 2 of the Golden" apple (Fig. 5b) and day 3 of the Granny Smith apple (Fig. 7b). We believe that no deeper meaning can be attached to these events than the effect of an unexpected systematic error.

## Discussion

The first part of the current research describes an experimental study of the detection of ethylene and ethanol released by Golden, Gala, Granny Smith, and Starkrimson apples using laser photoacoustic spectroscopy. This technique is very precise allowing that each gas to be measured with high efficiency. In a complex mixture of gases a proper absorption laser line must be chosen for the determination of concentration so that there is little or no interferences with other gases^35–40^. CO_2_ laser photoacoustic spectroscopy was used in the detection of ethylene and ethanol from apples and affords a better understanding of internal fruit development in storage conditions. These volatiles have important roles in the chemical processes taking place inside apples. The concentration of different volatile substances produced inside the apple fruits is determined by the permeability of the tissue, i.e. by the morphology of the apple surface and by its anatomical characteristics. These characteristics influence species variability, gas diffusion and various metabolic processes. An important role in the process of individual ripening of apples, ripening rate, qualitative changes, firmness, shelf life, the ideal storage requirement, is the internal atmosphere, i.e. the difference in the internal atmosphere of apples^5^. A considerable number of investigations have been conducted on the specific properties of the ageing and ripening of fruit^41–46^. Burg and Burg^47^ show that, in fruits gas diffusion follows Fick's law, which means that the flux of a gas, diffusing normally through a barrier, is dependent on the diffusion coefficient and concentration gradient. According to Kader and Morris^48^ anatomical features and not changes in biochemical pathways are responsible for differences in diffusion resistance.

In a study conducted by Bai et al.^49^ on Granny Smith apples, they shown that this variety of apples have relatively very few open pores (lenticels) in their peel surface and therefore they present a low rates of gas exchange. Gran and Beaudry^50^ studied the anaerobic respiration in three apple variety (Red Delicious, Red Fuji and McIntosh) and they observed wide variability in anaerobic respiration, which means that the products of anaerobic respiration such as acetaldehyde, ethylene and ethanol are influenced by the apple variety and storage condition.

The process of fruit ripening is influenced by the volatile substances accumulated or released differentially. Mendoza et al.^51^ studied apple tissue and showed that levels of volatile substances in apple fruits are associated with internal browning.

In our study we can observe that different varieties of apples have significant differences in their lives but as a general rule we can observe that while ethylene decreases ethanol increases with values depending on the variety. During 35-day trials, apples ethylene emissions manifested an almost constant emission in the first 14 days and then begin to fall, while the ethanol production of apples increased gradually. The Golden and Gala varieties showed the same behavior of the variation of the ethylene concentration and the highest values of ethylene at the beginning of the measurements. Golden and Gala apples presented the low emission of ethanol. At room temperature conditions, Golden and Gala apples became wrinkled and small brown patches appeared after only 7 days.

Compared to them, Granny Smith and Starkrimson varieties recorded lower values of ethylene concentration from the beginning of the measurements. These varieties withstood more at room temperature starting to present wrinkles and brown patches after 14 days. The Starkrimson apple variety had the lowest values of ethylene concentration, but the highest values of ethanol concentration, which means that after harvesting the ripening process stagnates and begins the process of fermentation which is very low in the absence of ethylene.

All apple varieties recorded a greater decrease in ethylene concentration on day 21 compared to the first 14 days, followed by a slight decrease in ethylene from day 21 to day 35. Starting with day 28 we can say that ethylene molecule was almost completely repressed meaning that the respiration of apples became very slow. The apples continued to degrade presenting lower weight, several spots brown patches and more wrinkles.

From a practical point of view, information about ethylene biosynthesis and ethanol action in the internal apple atmosphere reported in this paper can be used to create effective tools capable of evaluating and predicting the installation and evolution of the degradation process.

External defects on apples were investigated using a multispectral imaging system based on spectral filters chosen so that to cover the entire visible spectrum. Some were narrow-band filters (F460, F532 and F632) and others were wide-band (FBlue, OG2, OG3, RG6, BG12). The analysis of the histograms and of the global plots showed no clear patterns of change that could not be attributed to statistical or systematic errors, except in the case of the Granny Smith variety, which was studied for a longer period of time. In this case the histogram maxima moved toward left, toward darker tones, and the tops of the histograms were slightly flattened meaning that with the passage of time, the reflectivity of the apple decreased. Therefore we can say that the reflectivity of the apple decreased, before the degradation process was be noticeable to the naked eye.

Detection of defects on apples is important for an automatic apple sorting system and storage maintenance. Present results show promise that multispectral imaging may be used for detection of defects in apples.

CO_2_ laser photoacoustic spectroscopy and multispectral imaging can be associated to reveal when the degradation process begins in order to increase efficiency in the verification of fruit quality. These methods have been developed as a tool for non-destructive evaluation of fruit that could reduce postharvest losses during marketing by preventing deterioration and improve the final overall quality of commodities.

## Methods

### ***CO***_***2***_*** laser photoacoustic spectroscopy***

The first method that we used to measure internal gas concentrations was laser photoacoustic spectroscopy. This calorimetric technique measures with precision the number of molecules using the relation between the absorption and the amplitude of an acoustic signal. More specifically, the laser radiation is modulated at a wavelength that overlaps with the target speciesspectral characteristic and is then guided to a gas-filled PA cell. The gas molecules selectively absorb the incident radiation inside the PA cell, being excited at vibrational–rotational rates. The laser beam absorbs an amount of energy-dependent on the absorption coefficient, which is a function of pressure^29,32^. In LPAS, when the absorption coefficient is settled the response of the acoustic detector is independent of the electromagnetic radiation wavelength obtaining extremely low detection limits, which makes it possible detection of trace constituents in the sub-ppbV range. The nonradiative deactivation of the excited molecules via collisions produces periodical local heating (at the modulation frequency) and generates an acoustic wave recorded by microphones.

LPAS system used in this study incorporates as radiation source, a continuous wave (cw), line-tunable and frequency stabilized CO_2_ laser (LIR 25 SF) that emits radiation in the 9.2–10.8 μm region on 54 different vibrational–rotational lines with powers varying between 1 and 6.5 W depending on the emitted laser transition^52–58^.The laser is frequency stabilized to the center of the emitted line in terms of its output power versus frequency, with a typical instability of 3 × 10^−8^.All measurements were performed with the laser operating in the TEM_00_ mode for the selected wavelengths.

Another important element in the LPAS system is thePA cellwhere the gas of interest is detected when the laser radiation is absorbed by the specific molecules.The open resonant PA cellhas anH-type cylindrical geometry andis made of stainless steel and Teflon. Special attention is given to the PA cell in^29^. The main elements of the cell are anacoustic resonator (pipe), windows, gas inlets and outlets, microphones and acoustic filters to suppress the flow and window noise. In the tube wall, four Knowles electrets EK-23024 miniature microphones are mountedin series and generate a corresponding voltage for each detected acoustic wave.

The PA cell is connected to a complex gas handling system that controls the gas flow through the system and the purity of the cell.The gas handling system also includes two gas flow controllers, the sample cuvette and the potassium hydroxide (KOH) scrubber.

The process of the detection of the gas of interest is presented next. The laser beam is amplitude modulated by a mechanical chopper (DigiRad C-980) working at 564 Hz, focused by a ZnSe lens and introduced in the PA cell. The acoustic waves produced in the PA cell are detected with the four miniature microphonesKnowles electrets EK-23024 (sensitivity 20 mV/Pa each at 564 Hz) connected in series. For each detected acoustic wave the microphones convert the acoustics signal into an electrical signal. The electric signal is further fed into a phase-sensitive lock-in amplifier (Stanford Research System SR 830) operating at chopper frequency where the cell is excited at its first resonant longitudinal mode. The electrical signal is further fedto the lock-in amplifier where is analyzed and amplified so that the PA signal will be finally characterized by amplitude (voltage) and phase. The amplitude of the PA signal is linked to trace gas concentration while the phase expresses the vibrational–rotational relaxation time of the trace gas in a mixture. Because the measurements are dedicated to only one absorbing gas species, the phase of the acquired PA signal is fairly constant for all determinations. The phase is not bearing any information in this case, so it can be neglected. Therefore, our lock-in detection was performed near the zero-phase value. After passing through the PA cell, the laser beam power is measured by a powermeter (Laser Probe model RKT 30 CAL). The output signals of the lock-in amplifier and of the powermeter are introduced in the data acquisition interface (Keithley TestPoint software) module that automatically calculates the concentration. All experimental data are recorded in real-time and stored by a computer.The gas concentration defined by $c_{\min } = \frac{{V_{\min } }}{{\alpha P_{L} R}}$, where *c*_*min*_ (given in units of percent, ppmV, ppbV or pptV) is the minimum detectable concentration of a target trace gas that can be recorded based on the minimum measurable voltage signal *V*_*min*_ (V*)*achieved when the signal to ratio is unitary (SNR = 1), *R* (V cm/W) is the (voltage) responsivity of the PA cell or the calibration constant, *α* (cm^-1^ atm^-1^) represents the gas absorption coefficient at a given wavelength; and *P*_*L*_ (W) is the unchopped laser beam value.

In LPAS,when the absorption coefficient hassettledthe response of the acoustic detector is independent of the electromagnetic radiation wavelengthobtaining extremely low detection limits,which makes it possibledetection of trace constituents in the sub-ppbV range.

Our PA system is one of the most sensitive instruments having the quality factor of the system Q = 16.1, cell constant C = 5.41 × 10^3^ (Pa cm/W) cell, microphone responsivity 4 × 20x10^–3^ = 8 × 10^–2^ (V/Pa) and a minimum detectable concentration of 0.9 ppbV. The sensitivityis such that absorption smaller than10^–7^ cm^-1^ can be measured over path lengths of a few tens of centimeters. The small volume of the chamber makes it possible to accurately control the gas parameters, and the system can be operated either statically filled or in continuous gas flow mode.

LPAS was used to evaluate the ethylene and ethanol that are produced by Golden, Gala, Granny Smith and Starkrimson apples fruit in different stages of ripening. We investigated the physiological response of fruit only under aerobic conditions.

### Multispectral imaging

Multispectral imaging provides spectral analysis within specific wavelength ranges and has now been acknowledged as a simpler alternative to the hyperspectral imaging that supplies a continuous spectral analysis^59–63^. This method that was originally developed for space-born imaging, may capture light also from wavelengths outside of the visible spectral range, such as infrared and the ultraviolet. It can, therefore, provide information hidden to the human eye can capture. Most importantly, a quantitative rigorous analysis of the images may detect intensity and spectral patterns unnoticeable to the naked eye. Multispectral imaging can be used to address features such as ripening and external defects.

The experimental set-up is very simple: a Canon camera and a set of 8 filters placed between the camera and the sample (the apple).

The apple was placed in a box made of black cardboard in order to eliminate the ambient light and was placed every time in the same place. Moreover, DIC techniques based on FFT were used to digitally stabilize the image against slight movements of the camera or the apple. To illuminate the sample, we used a halogen lamp with a known transmission spectrum. The filters were mounted as close to the camera as possible. Picture were taken every day for a number of days.

**Figure 10:**
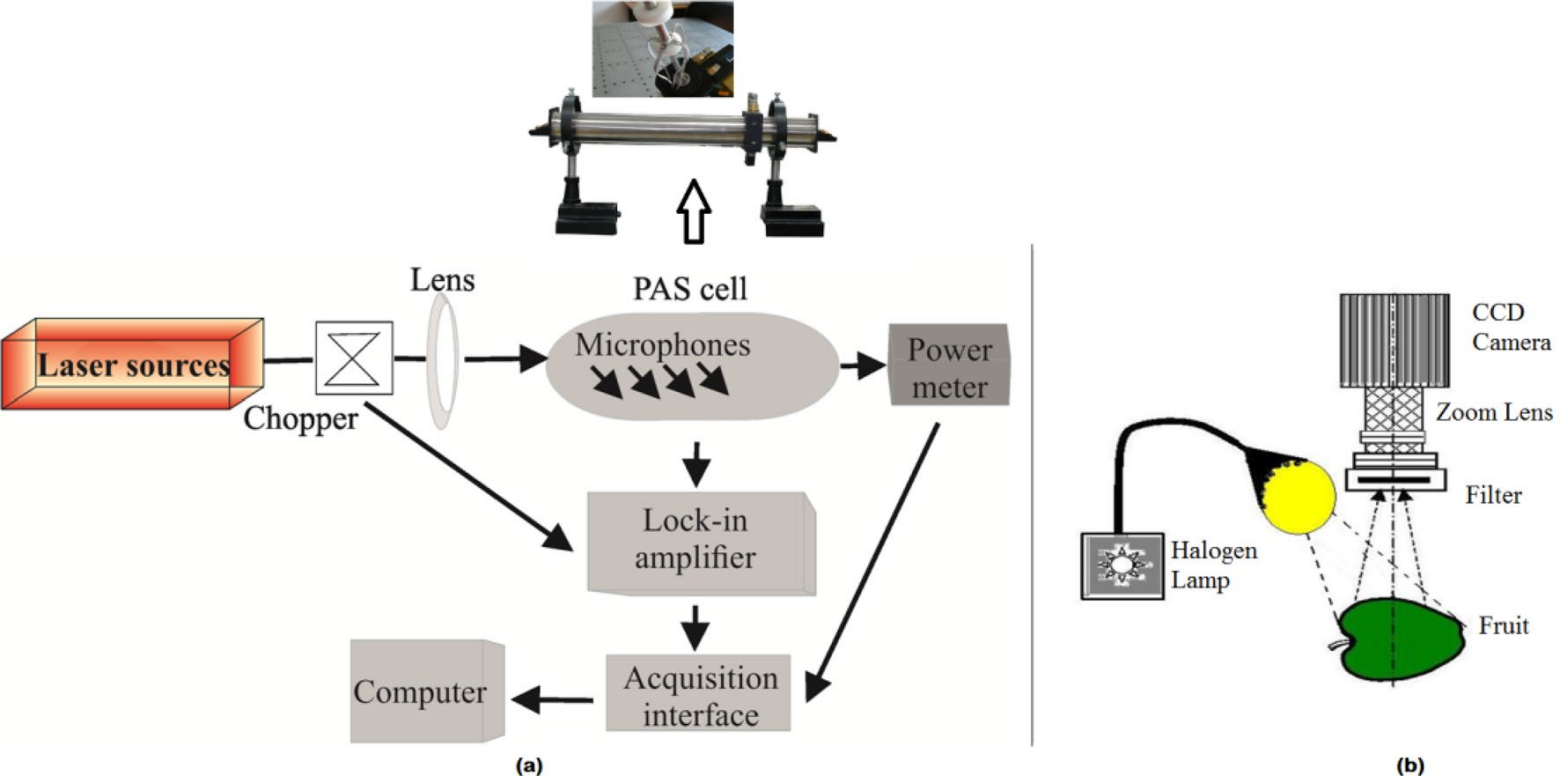
Experimental set-ups. (**a**) The block diagram of the CO_2_laser photoacoustic system. (**b**) The experimental set-up of multispectral imaging.

For non-destructive evaluation of Golden, Gala, Granny Smith and Starkrimson apple LPAS and multispectral imaging (see Fig. 10) are used to determine the internal volatiles of apples and to monitor their image and spectrum providing important information for fruit shelf life.

## Conclusion

CO_2_ laser photoacoustic spectroscopy and multispectral imaging were used as non-invasive methods to give us information about the internal and external damages produced in apples stored over a long period of time. The CO_2_ laser photoacoustic spectroscopy was used to measure the ethylene and ethanol concentration from the internal atmosphere and multispectral imaging to evaluate external defects of apples.

Using CO_2_ laser photoacoustic spectroscopy it was found that while ethylene decreases, ethanol increases with values depending on the variety. As a general rule, it can be observed that the high production of ethylene in the first days supports the appearance of degradation process and the increase of the ethanol concentration leads in time to the inhibition of ethylene. The differences in gases concentration conducted in this study on apples variety are influenced by the internal metabolic processes. Thus, a better understanding of the emission concentrations of ethylene and ethanol in different varieties of apples will facilitate choosing the correct actions to prevent degradation. Assessment in the endogenous volatile compounds from the fruit may demonstrate that the quality and quantity of volatiles may be linked to postharvest management of fruit.

The external defects on apples were investigated using a multispectral imaging system by monitoring the evolution in time of the histograms of the images corresponding to the various filters.

Making a connection between the reflectivity of the apples and the ethanol and ethylene hormone we can conclude that, in time, the ratio between the reflectivity of the apple and the value of the ethanol is inversely proportional and direct proportional with ethylene hormone. As the reflectivity decreases the value of the ethylene also decreases and the value of the ethanol increases because the apple is slowly degrading.

This work is the first attempt at bringing together CO_2_ laser photoacoustic spectroscopy and multispectral imaging that have shown some unique advantages and capabilities for evaluating internal atmosphere of apples and external defects. These two methods can be promising tools for assessing fruit quality by providing information about the degradation process, even when this process cannot be observed with the naked eye.

Exploring these new methods that can characterize both the internal and external aspects of fruits can have great potential to improve crop yield by delaying aging and maintaining fruit quality.
